# The Italian Validation of the Trust in Medical Researchers‐Short Scale: A Cross‐Sectional Study

**DOI:** 10.1002/hsr2.72480

**Published:** 2026-05-05

**Authors:** Stefano Ardenghi, Federico Zorzi, Laura Montelisciani, Marco Bani, Selena Russo, Giulia Rampoldi, Laura Antolini, Maria Grazia Strepparava

**Affiliations:** ^1^ School of Medicine and Surgery University of Milano‐Bicocca Monza Italy; ^2^ Fondazione IRCCS San Gerardo dei Tintori Monza Italy

**Keywords:** trust, trust in medical researchers, vaccine hesitancy, validation

## Abstract

**Background and Aims:**

Trust in medical researchers plays a critical role in orienting health‐related attitudes and behaviors, which profoundly influence healthcare outcomes, including research participation. This study aimed to contribute to the assessment of this construct by translating and validating the Italian version of the self‐administered questionnaire, Trust in Medical Researchers‐Short Scale (TMR‐SS).

**Methods:**

In March 2022, we invited participants from an existing longitudinal cohort to complete an online survey, including socio‐demographics, the General Trust Scale (GTS), the Vaccine Hesitancy Scale (VHS), the Edelman Trust in Pharmaceutics Barometer (ETPB), and the Italian back‐translated version of the TMR‐SS.

**Results:**

Confirmatory Factor Analyses confirmed the unidimensional structure of the original questionnaire, with good to excellent fit indexes. All the items in the original version were retained in the Italian adaptation. Both Cronbach's Alpha and McDonald's Omega indicated acceptable reliability. The Multi‐Group Confirmatory Factor Analysis indicated that the model was fully invariant across gender. There were no significant associations between the TMR‐SS and socio‐demographic variables. TMR‐SS inversely correlated with the VHS dimensions and positively correlated with the ETPB. There was no significant correlation between the TMR‐SS and the GTS.

**Conclusion:**

The Italian version of the TMR‐SS proved to be a valid, reliable, and easy‐to‐administer self‐report questionnaire of trust in medical researchers within the Italian context.

## 1 Introduction

Trust in medical research plays a critical role in shaping health‐related attitudes and behaviors, which profoundly influence healthcare outcomes [1, 2]. Furthermore, progress in medical research requires people's participation, and lack of trust is one of the most significant obstacles to this engagement [3]. For example, during the SARS‐CoV‐2 pandemic, the research pointed out that trust—both in government policies and in scientific research—was one of the most important factors influencing the likelihood of receiving the vaccine for SARS‐CoV‐2 [4]. Moreover, lack of trust in medical researchers was strongly associated with sociodemographic characteristics such as lower education, lower income, and belonging to an ethnic minority, thereby reinforcing social inequalities in vaccination practices [5].

As the products of medical research can profoundly impact public health and health‐related behaviors, building and maintaining trust in science and community engagement is paramount for healthcare systems and welfare policies to be universally effective [6, 7]. This cannot be achieved without increasing the investigation into trust in medical researchers itself. As a recent review highlighted, maintaining trust is crucial for researchers, health systems, and policymakers, and it advocated for a deeper exploration into the definition, assessment, and innovative insights derived from studying this construct [8].

Two validated scales for measuring trust in medical researchers are available: the Trust in Medical Researchers (TMR) [9] and the Trust in Medical Researchers Scale (TMRS) [10]. The two scales consider similar aspects and have been used in similar populations [11]. The TMR emphasizes trust in terms of safety, honesty, fidelity, and system trust [9], whereas the TMRS focuses on honesty, communication, and fairness, specifically addressing trust within minority groups [10]. The TMR offers the advantage of having a validated Short Scale (TMR‐SS) consisting of 4 items [9] which makes it easier to administer compared to the other questionnaire (12 items). When gathering research data, it is of paramount importance to administer agile assessment tools that minimize the respondent burden and maximize the likelihood of participation [12]. The present study contributed to the endeavor to increase knowledge of trust in medical researchers by translating and validating the Italian version of the self‐administered questionnaire, Trust in Medical Researchers‐Short Scale (TMR‐SS) [9, 13].

## 2 Methods

### 2.1 Procedures

As part of a wider longitudinal study on the impact of SARS‐CoV‐2 on Italian citizens [14], in March 2022, participants were invited to complete a survey, including measures about the perception of the pandemic severity, well‐being, and vaccination propension. Ethical approval for the study was received by the Ethical Committee of the University of Milano‐Bicocca, Italy (study n° 529 Prot. 0024533/20).

Eligibility criteria included being 18 or older, being proficient in Italian, residing in Italy at the time of the survey, and being able to provide informed consent. A convenience sample was recruited using a snowballing strategy; an invitation email describing the study and a link to the online survey was sent to students at Milano‐Bicocca University, who were asked to share the invitation with their social networks. Electronic consent was obtained from all participants.

### 2.2 Measures

Participants completed an online survey, including socio‐demographic and the following measures:

The General Trust Scale (GTS) [15] is a 6‐item questionnaire measuring participants' beliefs about the honesty and trustworthiness of others, in general. Items (e.g., “Most people are trustworthy”) are rated on a 5‐point Likert scale ranging from “1 = strongly disagree” to “5 = strongly agree.” Greater scores indicate higher levels of general trust. In this study, the unidimensional model of GTS was confirmed and revealed good fit indices: *χ* ^2^ = 16.31, *p* = 0.061, TLI = 0.988, CFI = 0.993, RMSEA = 0.045, SRMR = 0.022. The Cronbach's Alpha and McDonald's Omega coefficients of GTS were 0.835 and 0.846, respectively.

Five items of the Vaccine Hesitancy Scale (VHS) [16] and 2 items of the Vaccination Confidence Scale (VCS) [17] were used to assess attitudes toward vaccines. Considering the lack of specific tools to assess attitudes toward vaccines in adults, the selected items were adapted by changing the wording to focus on adults (e.g., “Childhood vaccines are effective” was changed to “Vaccines are effective”). The two factors resulting from the factor analysis were Lack of Confidence (VHS‐confidence), which included 4 items (e.g., “Vaccines are safe”) and Risks (VHS‐risks), which included 3 items (e.g., “New vaccines carry more risks than older vaccines”). Items are rated on a 5‐point Likert scale ranging from “1 = strongly disagree” to “5 = strongly agree.” Greater scores indicate higher levels of vaccine hesitancy. In this study, the two‐dimensional model underlying the VHS was confirmed and revealed good fit indices: *χ* ^2^_11_ = 27.18, *p* = 0.004, TLI = 0.980, CFI = 0.990, RMSEA = 0.061, SRMR = 0.034. The Cronbach's Alpha and McDonald's Omega coefficients of VHS‐confidence were 0.905 and 0.887, respectively; the Cronbach's Alpha and McDonald's Omega coefficients of VHS‐risks were 0.715 and 0.709, respectively.

The Edelman Trust in Pharmaceutics Barometer (ETPB) [18] is part of the 2020 Edelman Trust Barometer and measures trust in the pharmaceutical industry. The ETPB includes 4 items that measure the perception of the ethicality of pharmaceutical companies. Items are scored on an 11‐point Likert scale from “0” (e.g., “Serves the interests of only certain groups of people”) to “10” (e.g., “Serves the interests of everyone equally and fairly”), with higher scores indicating greater trust in the pharmaceutical industry. In this study, the unidimensional model of the ETPB was confirmed and revealed good fit indices: *χ* ^2^ = 12.34, *p* = 0.002, TLI = 0.970, CFI = 0.990, RMSEA = 0.113, SRMR = 0.018. Although the RMSEA for ETPB was greater than 0.08, which is a concern, in models with a small degree of freedom like this one, the RMSEA can often falsely indicate a poor‐fitting model [19]. The Cronbach's Alpha and McDonald's Omega coefficients of ETPB were 0.899 and 0.901, respectively.

The Trust in Medical Researchers‐Short Scale (TMR‐SS) [9] is a 4‐item scale measuring the level of trust in medical researchers. Items are scored on a 5‐point Likert scale from “1 = strongly disagree” to “5 = strongly agree,” with higher scores indicating higher levels of trust in medical researchers. The English TMR‑SS was translated into Italian following standard forward–backward procedures. Two bilingual researchers independently produced forward translations. Discrepancies were reconciled in a consensus meeting. An independent professional translator performed a back‑translation into English. The back‑translation was compared with the original, and minor wording adjustments were made. The final Italian TMR‑SS is provided in the Appendix.

### 2.3 Statistical Analysis

In this study, continuous variables were expressed as mean and standard deviation, while categorical variables were presented as absolute and relative frequencies (%) of each category. Comparisons of quantitative variables between groups of participants were carried out using a *t*‐test, and associations between categorical variables were tested using the Chi‐squared test. For the TMR‐SS dimension, a unidimensional model was tested, in which all items were loaded onto a single latent factor [20]. This was accomplished through a CFA [21]. Outliers were detected and excluded by the CFA using the Mahalanobis' distance (set at *p* < 0.001) [22]. In the CFA, the most common fit indices were used to evaluate the ability of the model to reproduce the observed data: *χ* ^2^ (*p* > 0.05), Root Mean Square Error of Approximation (RMSEA) (acceptable if < 0.10, good if < 0.08, very good if < 0.05), Standardized Root Mean Square Residual (SRMR < 0.05), Comparative Fit Index (CFI > 0.90), and Tucker–Lewis Index (TLI > 0.90) [23]. After identifying the latent factor underlying the TMR‐SS dimension, we assessed its reliability using Cronbach's alpha and McDonald's Omega coefficients, and tested its convergent validity using the Average Variance Extracted (AVE) and the Composite Reliability (CR) measures. We then calculated the factor scores and assessed the associations between the dimensions using Pearson's correlation coefficient to explore convergent and divergent validity of the TMR‐SS. Moreover, to evaluate the invariance across gender groups a Multigroup Confirmatory Factor Analysis (MGCFA) was carried out. Analyses used R version 4.4.2.

## 3 Results

### 3.1 Participants

A total of 413 Italians participated in the survey (Table 1). Outliers for the TMR‐SS dimension were excluded from the sample, resulting in a final sample of 410 participants. This number represented a good sample size for the validation procedure [24]. Approximately 74% of the sample were women. The mean age of the sample was 37.4 years (s.d. = 14.67), ranging from 18 to 75 years. Only seven participants (2%) were foreigners.

**TABLE 1:** Demographic characteristics.

	*N*	(%)
Gender		
Female	305	74%
Male	106	26%
Nationality		
Italian	406	98%
Other	7	2%
Marital status		
Single	123	30%
Married	160	39%
De facto	107	26%
Divorced‐Widow	23	6%
Employment status		
Employee	318	79%
Unemployed	84	21%
Income status		
Low	92	27%
Medium	225	65%
High	28	8%
Do you live alone?		
Yes	49	12%
No, with my partner/children	271	66%
No, with my family of origin	78	19%
No, with roommates/other	15	4%
Do you have children?		
Yes	170	41%
No	243	59%
Education		
High school or less	63	26%
Bachelor degree	59	24%
Master degree or PhD	121	50%
	Mean	s.d.
Age	37.4	14.67

### 3.2 Confirmatory Factor Analyses

The hypothesis of unidimensionality for the TMR‐SS model was tested. The goodness‐of‐fit indexes revealed an excellent fit between the model and the empirical data (*χ* ^2^ = 4.55, *p* = 0.10, TLI = 0.978, CFI = 0.993, RMSEA = 0.056, SRMR = 0.022), confirming the hypothesis of a single latent factor. Most of the items, with the exception of the reverse‐scored item 3 (i.e., “Medical researchers treat people like guinea pigs”) (*λ* = 0.36), exhibited good saturations (*λ*s > 0.40): item 1 (i.e., “Doctors who do medical research care only about what is best for each patient”) had a *λ* factor loading of 0.65, item 2 (i.e., “Doctors tell their patients everything they need to know about being in a research study”) had a *λ* factor loading of 0.63, and item 4 (i.e., “I completely trust doctors who do medical research”) had a *λ* factor loading of 0.69.

### 3.3 Reliability, Convergent Validity, and Descriptive Statistics of TMR‐SS

The convergent validity of the measurement model was assessed using the CR and the AVE. A CR value of 0.74 was considered satisfactory, even with the low number of items [25]. The AVE value for the TMR‐SS factor was 0.43. An AVE value less than 0.50 indicates that the error in the items is greater than the variance explained by the construct. This issue may be due to the low number of items in the latent factor. The Cronbach's Alpha and McDonald's Omega coefficients were appropriate (0.733 and 0.774, respectively). The means of TMR‐SS by demographic characteristics are presented in Table 2. No significant differences in the answer distributions of the TMR‐SS factor emerged for these characteristics, supporting its empirical adoption.

**TABLE 2:** TMR‐SS dimension means by demographic characteristics (*N* = 413).

	TMR‐SS		
	Mean	s.d.	*p*
Gender			0.14
Female	3.79	0.67	
Male	3.90	0.69	
Nationality			0.38
Italian	3.81	0.68	
Other	4.04	0.49	
Marital status			0.34
Single	3.89	0.65	
Married	3.75	0.70	
De facto	3.83	0.68	
Divorced‐Widow	3.73	0.57	
Employment status			0.70
Employee	3.81	0.67	
Unemployed	3.84	0.69	
Income status			0.72
Low	3.75	0.76	
Medium	3.81	0.67	
High	3.84	0.59	
Do you live alone?			0.08
Yes	3.76	0.66	
No, with my partner/children	3.78	0.68	
No, with my family of origin	3.99	0.66	
No, with roommates/other	3.70	0.63	
Do you have children?			0.38
Yes	3.78	0.63	
No	3.84	0.71	
Education			0.27
High school or less	3.81	0.68	
Bachelor degree	3.97	0.65	
Master degree or PhD	3.79	0.75	

**FIGURE 1:**
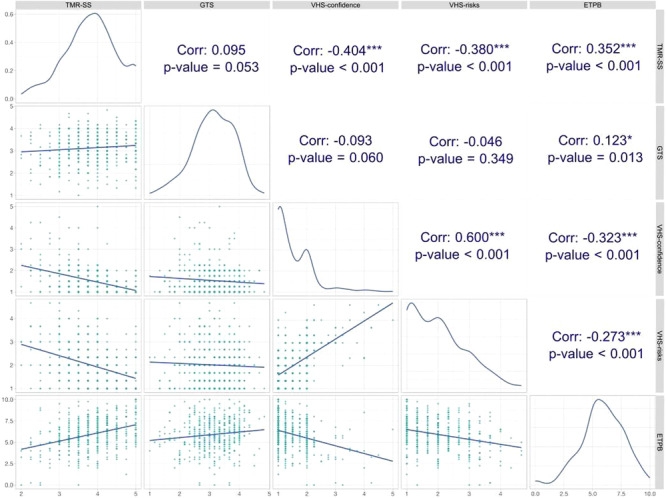
The Pearson correlation coefficients between the TMR‑SS factor scores and other scales. Bars show point estimates and 95% confidence intervals. VHS‑confidence and VHS‑risks are subscales of vaccine hesitancy. ETPB is Edelman Trust in Pharmaceutics Barometer. Significance levels: *p* < 0.001 indicated by *. Correlations computed using Pearson's *r* two‑tailed tests.

As shown in Figure 1, the TMR‐SS dimension was found to be statistically significantly associated with all other scales, except for the GTS (*r* = 0.095, *p* = 0.05). TMR‐SS was negatively associated with the two latent factors underlying the VHS dimension (*r*_VHS‐confidence_ = −0.404, *p* < 0.001; *r*_VHS‐risks_ = −0.380, *p* < 0.001) and positively associated with ETPB (*r* = 0.352, *p* < 0.001).

Multi‐Group Confirmatory Analysis (MGCFA) is a widely used method for conducting measurement invariance analysis [26]. To provide additional information about the TMR‐SS dimension, the unidimensional model was taken as the reference model for testing factorial invariance across gender. Testing for measurement invariance involves comparing a series of nested models with increasingly restrictive parameter specifications across groups [27, 28, 29]. Five levels of invariance were evaluated: configural, weak, strong, exact, and full. Measurement equivalence across gender was rejected if the Δ*χ* ^2^ between the two models (baseline vs. nested model) was statistically significant. The baseline model provided a plausible starting point for the study of factorial invariance, given that the number of factors is the same in both groups. The weak model adds factor loading invariance to the baseline model. The *p*‐value presented in Table 3 was not significant (*p* = 0.61), confirming weak invariance across groups. The strong model adds invariance of the latent intercepts to the weak model. The *p*‐value was also not significant (*p* = 0.18), indicating that strong measurement equivalence across gender was confirmed. The exact model adds residual invariance to the weak model. The *p*‐value was not significant (*p* = 0.12), confirming measurement exact equivalence across gender. The full model adds invariance of the factor means to the exact model. The Chi‐square difference was not significant (*p* = 0.06), indicating that the null hypothesis of full invariance across gender was supported. The key finding of MGCFA (Table 3) was that the TMR‐SS model should be considered completely invariant between males and females. This means that both groups share the same pattern of fixed saturation loadings, intercept values, and factor means.

**TABLE 3:** MGCFA of TMR‐SS structure on the full sample (*N* = 413): model invariance between males and females.

	*χ* ^2^	df	∆ df	∆ CFI	∆ TLI	∆ RMSEA	*p*
Configural invariance	5.903	4		NA	NA	NA	NA
Weak invariance	7.729	7	3	0.003	0.013	−0.025	0.61
Strong invariance	11.582	10	3	−0.005	−0.005	0.344	0.18
Exact invariance	21.354	14	4	−0.009	−0.005	−0.324	0.12
Full invariance	24.851	15	1	−0.008	−0.005	0.007	0.06

Abbreviations: CFI, Comparative Fit Index; df, degree of freedom; RMSEA, Root Mean Square Error of Approximation; TLI, Tucker–Lewis Index.

## 4 Discussion

This study reported the validation of the Italian version of the Trust in Medical Research‐Short Scale (Hall et al., 2006). The results of the Confirmatory Factor Analyses established the unidimensional structure of the original questionnaire, with good to excellent fit indexes. All the items in the original version were retained in the Italian adaptation. Both Cronbach's Alpha and McDonald's Omega reliability coefficients were acceptable, with values comparable to those of the original test. Additionally, the Multi‐Group Confirmatory Factor Analysis indicated that the TMR‐SS model was fully invariant across gender, therefore indicating that the instrument assesses trust in medical researchers identically for males and females.

The results did not show any significant associations of the TMR‐SS with socio‐demographic variables. This finding contrasts with previous research conducted in the United States of America, which identified socio‐demographic characteristics such as age, ethnicity, and income as predictors of trust in medical research (Hall et al., 2006; Smirnoff et al., 2018) and the propensity toward SARS‐CoV‐2 vaccine (Szilagyi et al., 2021). Conversely, other studies outside the US have not confirmed these associations [30, 31], suggesting that cultural differences could influence trust in medical research. Several explanations may account for discrepancies with previous studies, including sampling characteristics, measurement differences, statistical power, and temporal context. Cultural differences remain a possible factor, but our data do not allow us to test this hypothesis statistically, as cultural subgroups were too small to perform multigroup analyses.

As for the association with other measures of trust, TMR‐SS was inversely associated with vaccine hesitancy and positively correlated with trust in the pharmaceutical industry. This finding underscores the critical role of building trust in promoting healthcare access and delivery, which was particularly evident during the SARS‐CoV‐2 pandemic [32, 33, 34]. Conversely, trust in medical research did not significantly correlate with the measure of general trust in others. This is consistent with the literature, which indicates that general trust and particularized or categorical trust are only partially overlapping constructs [35, 36].

### 4.1 Study Limitations

While confirming that the Italian adaptation of the TMR‐SS has psychometric properties comparable to the original version, this study has several limitations that need to be addressed.

Although the sample was predominantly female (74%), measurement invariance testing through MGCFA demonstrated that the TMR‑SS operates equivalently across gender groups (see Table 3). Ethnic diversity is limited to 2% of the sample; therefore, measurement invariance across cultural subgroups could not be tested statistically. Nonetheless, this imbalance may limit generalizability. Second, the results of this study could not clarify the validity issues of the original tool. This is likely due to the fuzziness of the characterization of the measured construct, as highlighted in a recent review (Taylor et al., 2023). As such, further validation studies are needed to refine the definition of the construct of trust in medical research, thereby contributing to the development of more accurate measurement tools. Finally, the sample was drawn from a longitudinal cohort and may not be representative of the general Italian population.

## 5 Conclusion

The Italian version of the TMR‐SS has demonstrated acceptable validity and adequate reliability. Trust in medical researchers can be considered a measurable single‐factor construct in the Italian context. Although this study has some limitations, it provides a solid foundation for further research and cross‐national comparisons. Nonetheless, the Italian TMR‐SS proved to be a valuable instrument for assessing trust in medical researchers among the Italian population, which is crucial for fostering public engagement and improving healthcare delivery.

## Author Contributions

**Stefano Ardenghi:** conceptualization, writing – review and editing, writing – original draft, investigation, methodology. **Federico Zorzi:** writing – review and editing, conceptualization, writing – original draft. **Laura Montelisciani:** data analysis, writing – review and editing, writing – original draft. **Marco Bani:** conceptualization, writing – original draft, writing – review and editing, methodology, project administration, supervision, investigation. **Selena Russo:** conceptualization, writing – review and editing, writing – original draft, methodology. **Giulia Rampoldi:** writing – review and editing, writing – original draft, conceptualization. **Laura Antolini:** data analysis, writing– review and editing, writing – original draft. **Maria Grazia Strepparava:** supervision, writing – review and editing, writing – original draft.

## Funding

The authors have nothing to report.

## Disclosure

The lead author Marco Bani affirms that this manuscript is an honest, accurate, and transparent account of the study being reported; that no important aspects of the study have been omitted; and that any discrepancies from the study as planned (and, if relevant, registered) have been explained.

## Ethics Statement

This study was performed in line with the principles of the Declaration of Helsinki. The questionnaire and methodology for this study were approved by the Human Research Ethics Committee of the University of Milano‐Bicocca (study n° 529 Prot. 0024533/20).

## Consent

Informed consent was obtained from all individual participants included in the study.

## Conflicts of Interest

The authors declare no conflicts of interest.

## Policy on Using ChatGPT and Similar AI Tools

During the preparation of this work, the authors used Grammarly in order to improve readability. After using this tool, the authors reviewed and edited the content as necessary and took full responsibility for the publication's content.

## Data Availability

The data that support the findings of this study are available from the corresponding author upon reasonable request.

## 1

English version

Trust in Medical Researchers Generally – Short Scale

(Hall MA, Camacho F, Lawlor JS, DePuy V, Sugarman J, Weinfurt K. Measuring trust in medical researchers. Med Care. 2006;44(11):1048–1053. doi:10.1097/01.mlr.0000228023.37087.cb)

Intro: “These questions refer to medical research. Sometimes patients are asked to be in a research study about a medical problem that they may, or may not, have themselves. For example, someone might want to find out how a new drug works for a disease you have. Or, other times, someone might want to test a new vaccine on people who are perfectly healthy. We want to know what you would think about situations like that.”

**Table:**

	Strongly disagree				Strongly agree
1. Doctors who do medical research care only about what is best for each patient	1	2	3	4	5
2. Doctors tell their patients everything they need to know about being in a research study	1	2	3	4	5
3. Medical researchers treat people like “guinea pigs”*	1	2	3	4	5
4. I completely trust doctors who do medical research	1	2	3	4	5

* reverse score.

Italian Version

TRUST IN MEDICAL RESEARCHERS GENERALLY – SHORT SCALE – IT

Queste domande si riferiscono alla ricerca medica. A volte ai pazienti viene chiesto di partecipare a uno studio di ricerca su un problema medico che possono avere, oppure no. Per esempio, qualcuno potrebbe voler scoprire come funziona un nuovo farmaco per una malattia di cui soffri. In altri casi, invece, qualcuno potrebbe voler testare un nuovo vaccino su persone perfettamente sane. Vogliamo sapere che cosa penseresti di situazioni come queste.

**Table:**

	Completamente in disaccordo				Completamente d'accordo
1. I medici che fanno ricerca medica si preoccupano solo di ciò che è meglio per ogni paziente	1	2	3	4	5
2. I medici dicono ai loro pazienti tutto ciò che devono sapere sulla partecipazione a uno studio di ricerca	1	2	3	4	5
3. I medici ricercatori trattano le persone come “topi di laboratorio”*	1	2	3	4	5
4. Mi fido completamente dei medici che fanno ricerca medica	1	2	3	4	5

*reverse score
